# Influence of surface treatment on roughness, fracture force, flexural strength, and dynamic loading of a 3D-printed crown and bridge material

**DOI:** 10.1007/s00784-025-06518-8

**Published:** 2025-08-30

**Authors:** Michael Benno Schmidt, Sebastian Hahnel, Angelika Rauch, Martin Rosentritt

**Affiliations:** Department of Prosthetic Dentistry, UKR University Hospital Regensburg, 93042 Regensburg, Germany

**Keywords:** Surface treatment, Roughness, Fracture force, Flexural strength, Dynamic loading, 3D-printing, Additive manufacturing

## Abstract

**Objectives:**

To investigate how surface treatment affects fracture force, flexural strength, and dynamic loading cycles until failure of 3D-printed restorations.

**Materials and methods:**

Specimens (7 groups; *n* = 8 per group) were 3D-printed from an acrylate-based crown and bridge material. After cleaning and post-polymerization, specimens were treated with either silicon carbide paper (1000 grit; 1000/4000 grit) or blasting (Al_2_O_3_; 1 bar/125 µm; 2 bar/125 µm; 1 bar/250 µm) to simulate laboratory treatment. Surface roughness (Arithmetic mean Sa/maximum roughness height Sz; ISO 25178-2); fracture force (FF) and biaxial flexural strength (BFS; ISO 6872) were determined. The number of dynamic load cycles (LC) to failure was determined under cyclic loading in a BFS staircase approach. Statistics: ANOVA, Bonferroni-test, Kaplan-Meier survival, Pearson correlation; α = 0.05.

**Results:**

BFS ranged between 94.4 MPa and 199.9 MPa, FF between 260.6 N and 428.6 N and Sa/Sz between 0.0/1.0 μm and 1.8/18.4 μm. BFS, FF and Sa/Sz showed significant differences between the treatments (*p* < 0.001) and individual groups (*p* ≤ 0.013). Mean LC ranged between 204,364 and 267,637 cycles. ANOVA comparisons (*p* = 0.706) and Log Rank test (Chi^2^: 10,835; *p* = 0.094; Fig. 2) revealed no significant differences between the loading cycles. Surface treatment with either silicon carbide papers or blasting protocols had a significant influence on FF, BFS, Sa, and Sz, but not on LC.

**Conclusions:**

Surface treatment affected the fracture force and biaxial fracture strength of a 3D-printed crown. It showed no influence on the long-term dynamic behavior.

**Clinical relevance:**

Smooth surfaces improve the stability of a restoration fabricated from 3D-printing resins. Extensive surface roughness treatment before cementation can reduce the stability of a crown.

## Introduction

Resin-based restorations can be easily and quickly processed with computer aided design (CAD) and computer aided manufacturing (CAM). 3D-printing of liquid photopolymer resins with digital light processing (DLP) vat 3D-printing systems [1, 2] require little equipment and feature low production costs in combination with simple handling [3]. Despite of limited mechanical properties, these restorations can be used as long-term temporary or permanent dentition [4]. 

For optimizing the properties of the restorations, it is essential to coordinate the 3D-printing techniques with the individual properties of the material [5], e.g. by matching printing speed and viscosity of the resin [6] or the cleaning process subsequent to printing in order to remove residual monomers [7]. Post-polymerisation is finally performed to provide restorations with sufficient polymerisation and strength. Summarizing, the properties of a restoration are dependent on both the material and the fabrication parameters (layer thickness, platform orientation, cleaning, post-polymerisation) [8]. 

In addition, surface quality plays an important role for strength and performance of a restoration. The conventional wisdom is that adequate adjustment or surface polishing as well as pre-treatments such as blasting prior to insertion may improve the clinical longevity of a restoration [9]. While damage and cracks generally reduce strength, polishing reduces roughness and improves stability [10]. Smooth surfaces do also reduce the risk of crack formation, development and propagation [11, 12], which is particularly well known for brittle materials such as composites and ceramics.

These considerations raise the question in how far surface treatments such as blasting or polishing can influence the strength and long-term performance of additively manufactured restorations. The hypothesis of this in-vitro study was that surface treatment affects fracture force, flexural strength, and loading cycles until failure of 3D-printed restorations.

## Materials and methods

Specimens (diameter 16 mm, height 2 mm; *n* = 8 per group) were additively manufactured with a P30 + DLP-printer (Straumann, Basel, CH) from an acrylate crown and bridge material (P Pro C&B A3 019.3068, Straumann; LOT: 224507 A). The printing direction was 90° with a layer thickness of 100 μm. After cleaning (Isopropanol 99%, Walter, Kiel, D; #UN1219; P Wash, Straumann; pre-cleaning 3:10 min, cleaning 2:20 min, drying 1:30 min; LOT:) and post-polymerisation (P Cure, Straumann; LED, 10 min, vacuum, UV–A: 400–315 nm; UV-B 315–280 nm, heating), specimens were sanded with either silicon carbide paper (ø 250 mm; 1000 grit LOT: OT3/40400227 or 1000/4000 grit LOT: AB1599039/40400232; Tegramin 25, Streurs, Kopenhagen, DK; 5 N, 20 s, 120 rpm, counter-rotating) or blasted (blasting medium: Aluminium oxide; 1 bar/125 µm or 2 bar/125 µm #: 75606 or 1 bar/250 µm #: 75250; PG400, Harnisch & Rieth, Winterbach, D; undulating, 5 racks, 2 times) to simulate laboratory treatment. Surface roughness (Sa/Sz) was measured using a confocal laser scanning microscope (VK-100, Keyence, Osaka, J) according to ISO 25178-2:2019 (50x; range = 7 mm, z-resolution = 0.005 μm, x-resolution = 0.01 μm, repeatability = 0.02–0.05 μm, ND-filter 100%).

### Biaxial flexural strength (BFS)

ISO piston-on-three-ball test [13] (Z2.0, Zwick/Roell, Ulm, D) was performed to evaluate biaxial flexural strength (BFS). Specimens were placed on a ring-shaped support bearing (THS1620, Grip-Engineering Thümler, Nürnberg, D) with the pre-treated surface facing downwards. The bearing consisted of three stainless steel spheres (Ø 3 mm), which were arranged in form of an equilateral triangle at 120° (diameter = 10 mm). The samples were positioned in the middle of the bearing. After applying a preload of 0.5 N, the load was applied centrally applied with a steel sphere (Ø 3 mm). The load was applied at a rate of one millimeter per minute (1 mm/min) by the piston, which had a diameter of 1.6 mm. A 0.05 mm polyethylene foil (1-7090, neoLab Migge, Heidelberg, D) was used to equalize force distribution. Fracture force (FF) was determined and BFS was calculated using the following equation:

1 $$\mathrm\sigma=-0.2387\mathrm P(\mathrm X\:-\:\mathrm Y)/\mathrm d2$$

Legend:

- σ = biaxial flexural strength (MPa);
- P = fracture force (N);
- d = sample thickness at fracture origin (mm).

The variables X and Y were determined as follows:

2 $$\mathrm X=(1\:+\:\mathrm v)\ln(\mathrm r2/\mathrm r3)2+\lbrack(1\:-\:\mathrm v)/2\rbrack\mathrm r22/\mathrm r3$$

3 $$\mathrm Y=(1\:+\:\mathrm v)\lbrack1\:+\:\ln(\mathrm r1/\mathrm r3)2\rbrack+(1\:-\:\mathrm v)(\mathrm r1/\mathrm r3)2$$

Legend:

- v = Poisson’s ratio (0.3);
- r_1_ = radius of the supporting bearing;
- r_2_ = radius of the loaded area;
- r_3_ = radius of the sample.

## Cycling load to failure

The number of load cycles (LC) to failure was determined under cyclic loading in a staircase approach. Specimens were positioned and loaded as in the BFS situation. In the stepwise loading test, each specimen was preloaded with 50 N and dynamic force was applied for 10^5^ loadings (f = 3 Hz) for each loading step, including (step 1) 50–100 N, (step 2) 50–150 N, (step 3) 50–200 N, (step 4) 50–250 N, and (step 5) 50–300 N. Tests were carried out in distilled water (37 °C) using a simulation unit for dynamic loading (F1000; Prematec, D). Fracture patterns were categorised into fractures with two, three, or four pieces.

## Statistics

Data were controlled for homogeneity using the Shapiro-Wilk test. Means and standard deviations were calculated and differences between means were analysed (Analysis of Variance ANOVA, Bonferroni). Strength and direction of a linear relationship between two continuous variables were investigated (Pearson correlation). Cumulated survival was calculated with Kaplan Maier Log Rank test ((Mantel-Cox; SPSS 29.0, IBM, Armonk, NY, USA; α = 0.05).

## Results

**Fig. 1:**
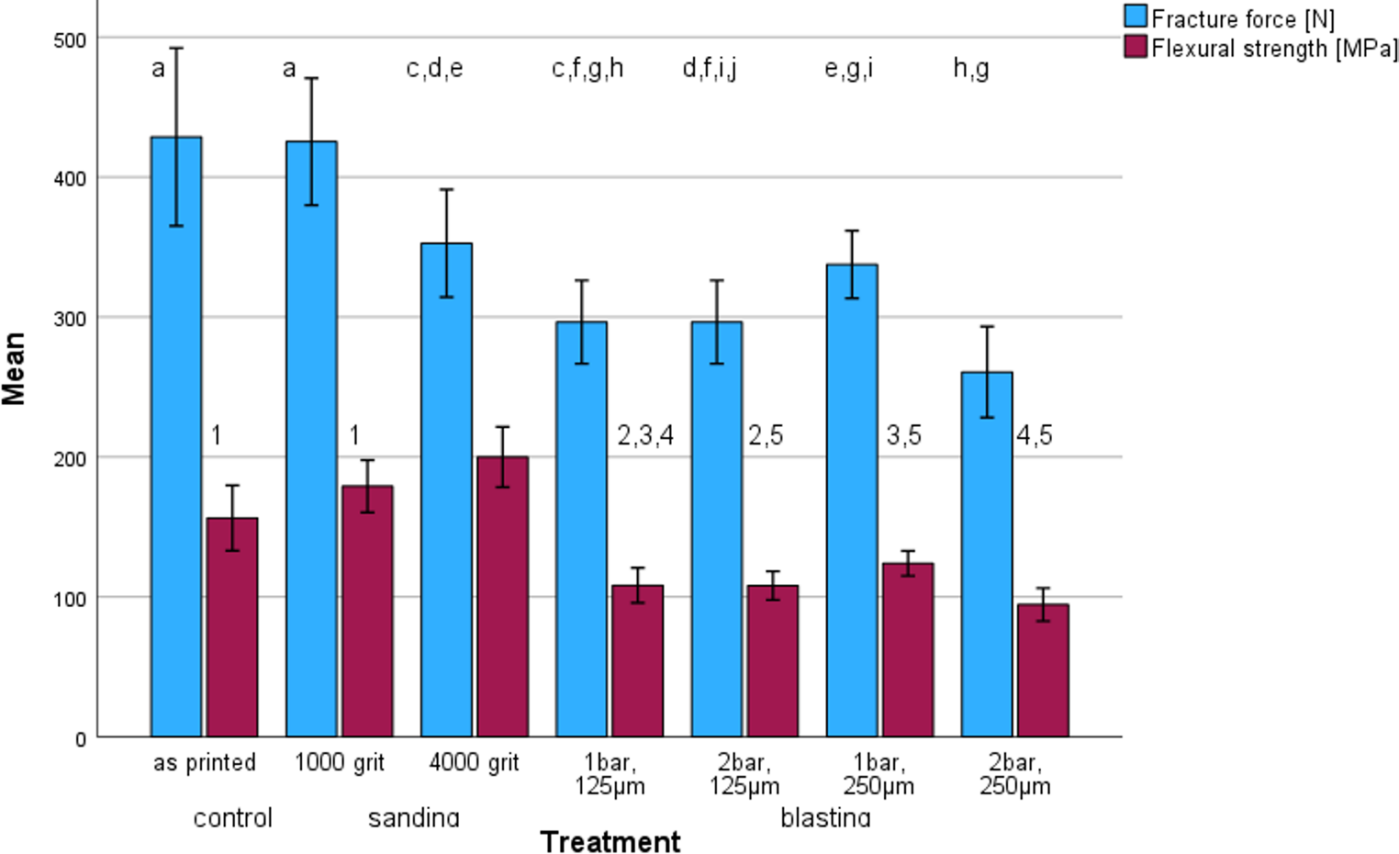
Fracture Force FF [N] (blue bars) and Flexural Strength FS [MPa] (red bars) (mean and standard deviation) depending on the surface treatment. (Identical letters indicate no significant differences for fracture force; identical numbers indicate no significant differences for flexural strength)

Figure 1 shows the mean biaxial flexural strength BFS. The values ranged between 94.4 (2 bar, 250 μm) and 199.9 MPa (4000 grit), with significant differences between the treatments (ANOVA: *p* < 0.001) and individual groups (*p* ≤ 0.014).

Mean fracture force FF (Fig. 1) varied between 260.6 N (2 bar, 250 μm) and 428.6 N (as printed), with significant differences between the treatments (ANOVA: *p* < 0.001) and individual groups (*p* ≤ 0.013).

**Fig. 2:**
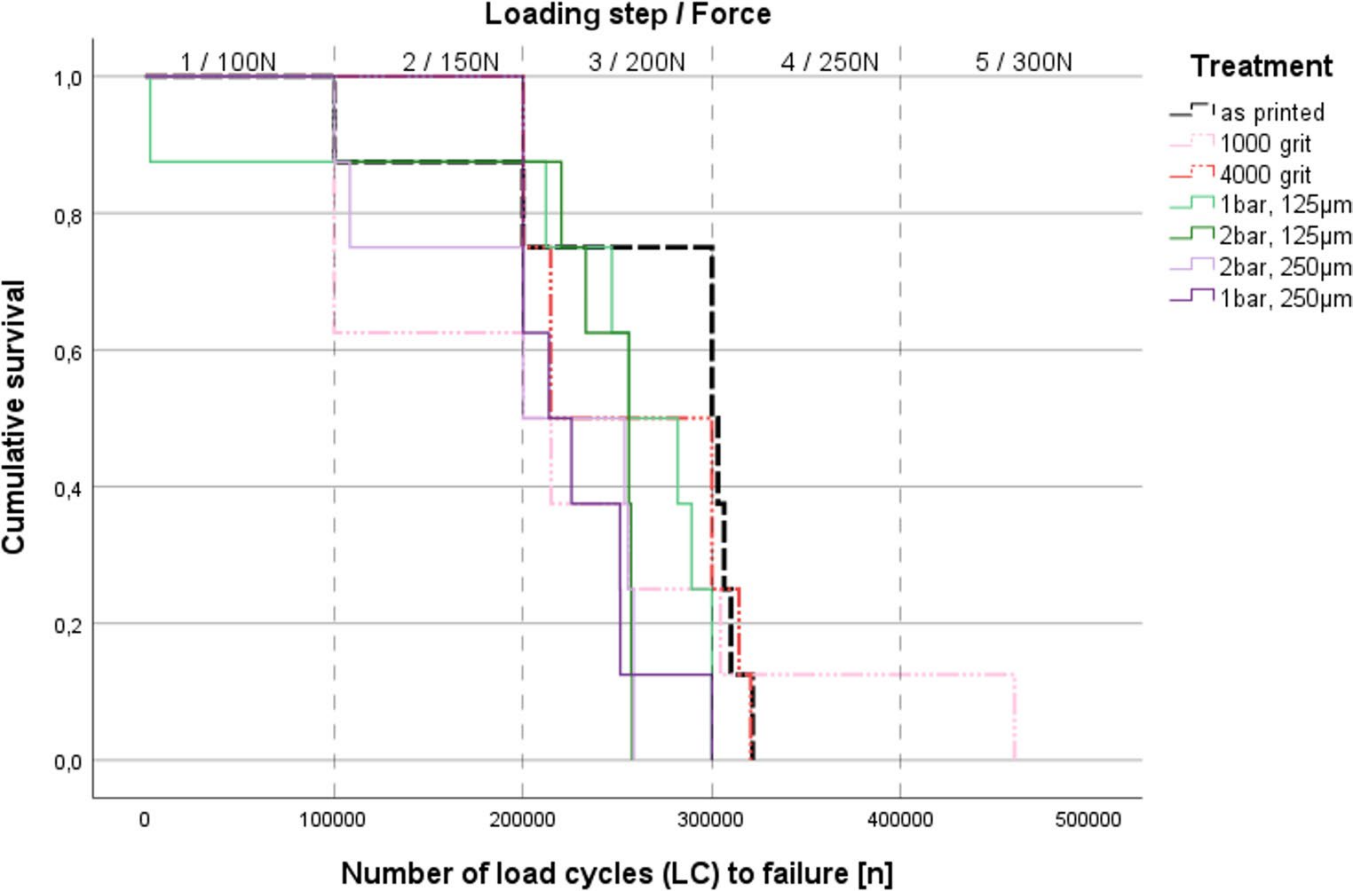
Cumulative survival versus number of load cycles (LC) to failure (Kaplan Meier Log Rank (Mantel-Cox) test (Chi^2^: 10,835; *p* = 0.094)) (Mean loading cycles to failure: i) as printed: 267000, ii) 1000 grit: 216000, iii) 4000 grit: 257000, iv) 1 bar/125µm: 236000, v) 2 bar/125µm: 229000, vi) 1 bar/250µm: 230000, vii) 2 bar/250µm: 204000.)

Figure 2 shows the mean load cycles to failure LC. Results between 204,364 (2 bar, 250 μm) and 267,637 (as printed) were found. ANOVA comparisons (*p* = 0.706) and Log Rank test (Chi^2^: 10,835; *p* = 0.094; Fig. 2) revealed no significant differences between the loading cycles.

For roughness (Table 1), mean Sa/Sz ranged between 0.0/1.0 μm (4000 grit) and 1.8/18.4 μm (2 bar, 250 μm), with significant differences between the treatments (ANOVA: *p* < 0.001) and individual groups (*p* < 0.001).

**Table 1:** Arithmetic mean Sa and maximum roughness height Sz (means, standard deviation) depending on the surface treatment (identical letters indicate significant differences to the “as printed” control; *p* ≤ 0.013)

		Roughness			
		Sa [µm]		Sz [µm]	
Treatment		Mean	Standard deviation	Mean	Standard deviation
Control	as printed	0.4 ^a, b, c, d,^	0.1	6.3 ^a, b, c, d, e^	2.6
Sanding	1000 grit	0.2	0.1	2.0	0.7
	4000 grit	0.0	0.0	1.0 ^a^	0.6
Blasting	1 bar, 125 μm	1.4^a^	0.4	14.1 ^b^	2.1
	1 bar, 250 μm	1.3^b^	0.6	14.0 ^c^	3.3
	2 bar, 125 μm	1.5^c^	0.5	16.8 ^d^	4.4
	2 bar, 250 μm	1.8^d^	0.5	18.4 ^e^	3.9

**Fig. 3:**
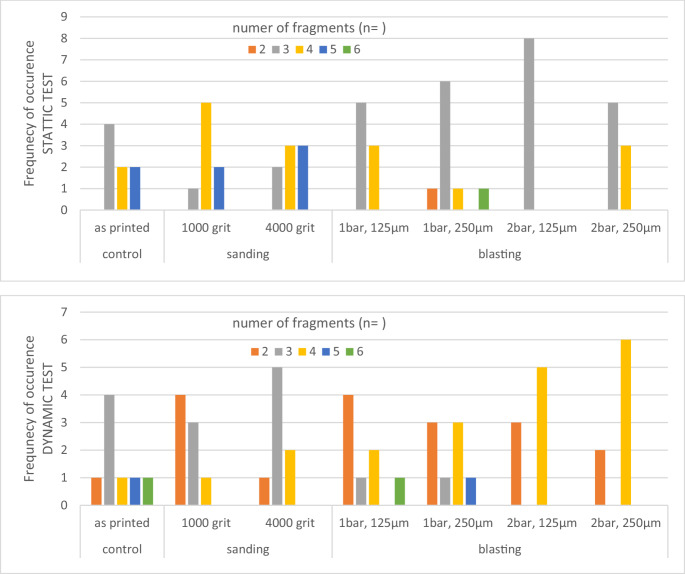
Frequency of occurrence for the different numbers of fragments (*n* = 2 to 6) after static failure test and during dynamic testing (Total sample size: 8). Above: static loading test, below dynamic loading test

Figure 3 shows the frequency of failure occurrence for the different numbers of fragments after static failure test and during dynamic testing. Number of fragments varied between two and six. In the static test, sanding had no influence on the fracture pattern, while blasting was found to reduce the number of fragments in three quarters of the cases. During the dynamic test, a shift in the fracture pattern towards a lower number of fragments was found compared to the static group. Sanding and low blasting pressure resulted in a greater diversity of fragments.

Surface treatment with either various silicon carbide papers or various blasting protocols had a significant influence on FF (Pearson: −0.705; *p* ≤ 0.001), BFS (−0.623; *p* ≤ 0.001), Sa (0.708; *p* ≤ 0.001), and Sz (0.710; *p* ≤ 0.001), but not on LC (−0.143; *p* = 0.294). FF and BFS showed a significant (> 0.663; *p* ≤ 0.001) correlation with Sa and Sz.

## Discussion

Data suggest partial acceptance of the research hypothesis. While fracture force and biaxial flexural strength were significantly influenced by surface treatment, loading cycles were not different.

Blasting reduced fracture force about 25–40%. While the blasting pressure in combination with the 125 μm blasting material had no effect, blasting with 250 μm blasting material caused a decline in fracture force. Surface roughness produced by blasting correlated with fracture forces. With increasing surface roughness, decreased fracture forces were identified. These results corroborate a previous study and indicate that higher Rz values foster fractures of a restoration [14]. However, this correlation is likely to be dependent on the individual material, with different surface roughness thresholds for each material and surface treatment [9]. The decline in fracture forces observed in highly polished specimens and those with reduced Rz values is, however, surprising: Polishing with 1000 grit produced no effects, but polishing with 4000 grit significantly reduced fracture forces despite of lower roughness. A similar contradiction could not be identified for flexural strength, which becomes clear by comparing changes in fracture force and biaxial fracture strength - i.e. compressive force in relation to specimen thickness. Polishing reduces the thickness of the specimen and consequently its fracture force. Biaxial fracture strength is increased by polishing as the surface becomes smoother, which indicates that smooth surfaces may reduce the risk of fracture by increasing strength, whereas a reduced wall thickness reduces the overall stability of the restoration [15]. These observations therefore have a direct impact on the clinical application that dentists and dental technicians should be aware of [4], as a restoration must have sufficient wall thickness after roughening for bonding as well as occlusal adjustment and polishing. The authors also observed the tendency that the different treatments affect the failure of restorations, as blasting reduced the number of fracture fragments (Fig. 3). This observation might be due to the more inhomogeneous surface resulting from blasting with one superficial defect.

Most failures during dynamic loading occurred in the range between 100,000 and 300,000 load cycles. The failure behaviour seemed to be independent of the treatment: Surprisingly, blasting and polishing in the tensile zone had no significant effect on the number of cycles until failure. This phenomenon could be related to the height of the selected forces and the number of loading steps applied, as only the last loading step was 300 N, which represents 85–140% of the maximum static fracture force. Only a few failures occurred at loads higher than 200 N, and most frequently failures occurred in the force range up to 150 N, which is well below the maximum fracture force of the individual specimens. This phenomenon indicates that the samples showed significantly less strength during dynamic loading than in the static test, which emphasizes the relevance of dynamic testing. In contrast to cleaning and post-polymerization, the influence of surface roughness on the performance of the specimens was only subordinate. On average, however, the specimens with the hardest processing protocol (blasting with 2 bar and 250 μm) yielded the shortest survival times and those without treatment (“as printed”) and those with finely polished surfaces yielded the longest survival times. Nevertheless, a correlation with Sz could not be confirmed, which indicates that surface treatment – as a simulator of a realistic clinical scenario – has no effect on the dynamic behavior of specimens manufactured from the printing resin. For dynamic loading, the fracture pattern of the samples appeared to be different from the static tests, as there was a trend towards fewer fracture fragments. Similar to the static tests, a small number of fracture fragments was found for the blasted specimens, indicating a more homogenous surface with reduced failure risk. However, if sample thickness was included in the Kaplan Meier analyses, a significant improvement in survival rate for specimens with smoother polishing was observed. In these cases, blasted surfaces showed no difference to the untreated surfaces (“as printed”).

As only one printing resin was investigated in the current study, the question arises whether the results can be easily transferred to other printing materials. Different behaviour might be attributed to individual material components such as methacrylate oligomers, methacrylate monomers, or photo-initiators [16], or crosslinking [17]. Furthermore, the results might be influenced by the alignment of the individual printing layers [18]. Post-curing increased hardness and the degree of conversion increased with post-curing time [19], and the flexural fatigue limit of resin-based systems is influenced by conversion [20, 21]. An increased light intensity during post-curing might cause embrittlement and, as a result, reduced flexural fatigue limits or flexural strength. Applied energy densities, the formation of internal stresses during curing, the monomer/polymer ratio as well as crosslinking may modify the final material and its mechanical behaviour, which may account for the lacking correlation between flexural fatigue limits and in-depth degree of conversion [20].

## Conclusions

Surface treatment has a significant effect on the fracture force and biaxial fracture strength of the restoration, but the effect was less imperative for the long-term dynamic behaviour. Smooth surfaces in combination with sufficient wall thickness may improve the stability and long-term performance of a restoration fabricated from printing resins.

## Data Availability

No datasets were generated or analysed during the current study.
